# Essential Dynamics Ensemble Docking for Structure-Based GPCR Drug Discovery

**DOI:** 10.3389/fmolb.2022.879212

**Published:** 2022-06-29

**Authors:** Kyle McKay, Nicholas B. Hamilton, Jacob M. Remington, Severin T. Schneebeli, Jianing Li

**Affiliations:** Department of Chemistry, University of Vermont, Burlington, VT, United States

**Keywords:** computer aided drug design, PAC1 receptor, antagonist, virtual screening, molecular dynamics, principal component analysis

## Abstract

The lack of biologically relevant protein structures can hinder rational design of small molecules to target G protein-coupled receptors (GPCRs). While ensemble docking using multiple models of the protein target is a promising technique for structure-based drug discovery, model clustering and selection still need further investigations to achieve both high accuracy and efficiency. In this work, we have developed an original ensemble docking approach, which identifies the most relevant conformations based on the essential dynamics of the protein pocket. This approach is applied to the study of small-molecule antagonists for the PAC1 receptor, a class B GPCR and a regulator of stress. As few as four representative PAC1 models are selected from simulations of a homology model and then used to screen three million compounds from the ZINC database and 23 experimentally validated compounds for PAC1 targeting. Our essential dynamics ensemble docking (EDED) approach can effectively reduce the number of false negatives in virtual screening and improve the accuracy to seek potent compounds. Given the cost and difficulties to determine membrane protein structures for all the relevant states, our methodology can be useful for future discovery of small molecules to target more other GPCRs, either with or without experimental structures.

## Introduction

**FIGURE 1:**
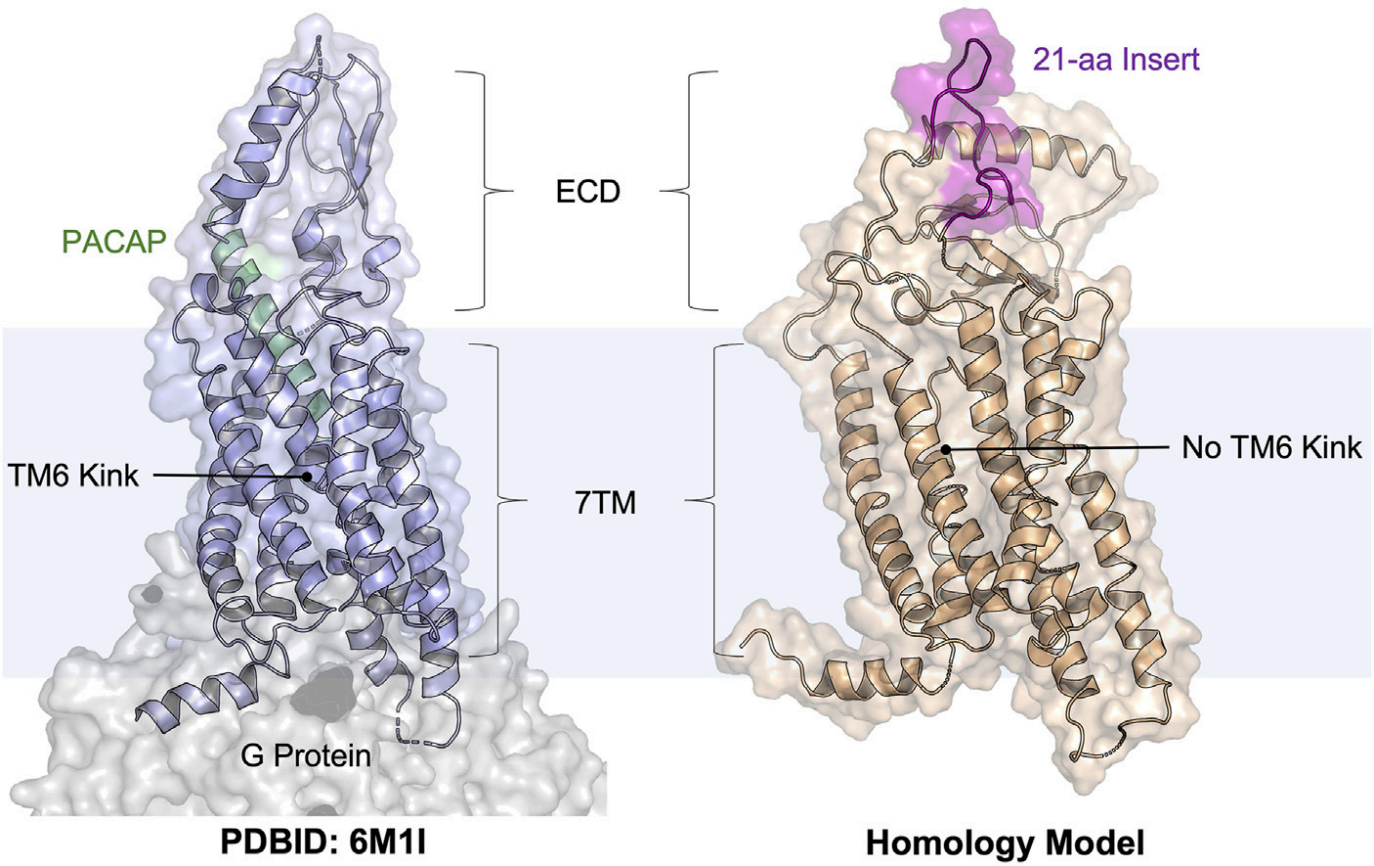
Cartoon illustrations of the PACAP-bound PAC1R model (PDBID: 6M1I, PAC1very short) and our homology model (template PDBID: 4L6R, PAC1null, simulation snapshot at 500 ns). The PAC1null isoform is more biomedically relevant than the very short isoform. The PACAP peptide is shown as a helix cartoon (pale green); the 21-amino acid ECD insert (see the sequence in Supplementary Figure S1) is shown as a flexible coil (purple). This study focused on docking to the peptide-binding pocket.

Many G protein-coupled receptors (GPCRs) are being investigated as important therapeutic targets, but the success rate of structure-based drug design (SBDD) for GPCRs remains to be further improved (Hauser et al., 2017; Wootten et al., 2018; Odoemelam et al., 2020). One of the primary challenges is that the three-dimensional (3D) structures of most GPCRs have not been fully determined. Even with latest breakthroughs in protein structure prediction like AlphaFold (Jumper et al., 2021), the available structures may not represent the conformational states needed for accurate SBDD. The receptor (*ADCYAP1R1*, hereafter referred to as PAC1R) of the pituitary adenylate cyclase-activating peptide (PACAP), an emerging therapeutic target for stress-related disorders (Hammack et al., 2009; Ressler et al., 2011; Roman et al., 2014; Missig et al., 2017; Liao et al., 2019a), is a good example. Currently, the full-length PAC1R structures in the Protein Data Bank (PDB) are short isoforms (Uniprot ID: P41586-3) (Kobayashi et al., 2020; Liang et al., 2020), but the structures of the most prevalent long isoforms—PAC1null (Uniprot ID: P41586) or PAC1hop (Uniprot ID: P41586-2) — are still unavailable (Liao et al., 2019b). All the published structures of PAC1R are complexed with peptide agonists and a heterotrimeric G protein complex (Figure 1), and thus do not represent the inactive conformations required for antagonist development. So far, it is thought that over 40% of GPCRs have more than one isoform (Marti-Solano et al., 2020), and each GPCR can adopt multiple conformational states which can be stabilized upon interactions with binding partners (Li et al., 2013; Vardy and Roth, 2013). For accurate SBDD, it is important to employ conformations of the most medically relevant isoform, as it is to this ensemble of 3D pocket structures that the drug must show affinity. Here, we used PAC1R as a model system and investigated how to improve modeling accuracy and to gain predictive power for SBDD with limited 3D structural information, using the method of Essential Dynamics Ensemble Docking (EDED). With the proof of principle, this method can be readily generalized to develop new therapeutic targets to target a wider range of GPCRs.

PAC1R and its endogenous peptide hormone PACAP play an important role in neural development, calcium homeostasis, glucose metabolism, circadian rhythm, thermoregulation, inflammation, feeding behavior, pain modulation, as well as stress, and related endocrine responses (Harmar et al., 2012; Bortolato et al., 2014; Culhane et al., 2015). For example, increased levels of PACAP in the blood have been reported in women diagnosed with post-traumatic stress disorder (Ressler et al., 2011), implicating chronic activation of the PAC1R in the disorder. Other studies (Boehr et al., 2009; Missig et al., 2017) have suggested that PAC1R activation mediates the adverse emotional consequences of chronic pain via downstream MAPK/ERK activation. Thus, these prior studies indicate that PAC1R antagonism, especially with small-molecule antagonists, represents a new strategy to treat stress, chronic pain, and related disorders (Ressler et al., 2011). Similar to other class B GPCRs, PAC1R possesses a heptahelical transmembrane domain (7TM) and an extracellular domain (ECD) (Odoemelam et al., 2020). Most of the neural and peripheral tissues known to date contain the PAC1null or PAC1hop isoforms that includes a 21-amino acid insert in the ECD (Figure 1), which is missing in available PAC1R structures in the PDB (May and Parsons, 2017). This ECD insert was found highly dynamic in our previous modeling studies (Liao et al., 2017; Liao et al., 2021), but its role in regulating PAC1R remains unknown. While PAC1R antagonists are being developed as potential treatments for stress-related disorders, the agonist-bound cryo-EM structures are not directly applicable to computational design or screening of PAC1R antagonists. GPCRs spontaneously adapt active and inactive signaling states, each of which are characterized by broad conformational ensembles. In the confirmational selection view, agonists and antagonists stabilize GPCR conformations of the active and inactive ensembles, respectively (Boehr et al., 2009; Abrol et al., 2013). It is now well accepted that to accurately design GPCR ligands as drug candidates, one should use active conformations for agonist design and inactive conformations for antagonist design. With the transition between active and inactive GPCR conformations occurring on the millisecond timescale (Vilardaga, 2010; Heyden et al., 2013; Weis and Kobilka, 2014; Scherer et al., 2015), it is computationally demanding to obtain the inactive PAC1R conformations from the agonist-bound cryo-EM structures via molecular dynamics (MD) simulations. Instead, we seek to use a homology model in this work and test with the EDED method.

Ensemble docking utilizes multiple receptor models for pocket sampling, obtained from clustering the conformations sampled by MD simulations for molecular docking, and displays noted improvement at identifying GPCR ligands when compared to docking against a single experimental structure (Lin et al., 2002; Huang and Zou, 2007; Amaro et al., 2018; Velazquez et al., 2018; Li et al., 2019; Acharya et al., 2020; Bhattarai et al., 2020; Chandak et al., 2020; Jukič et al., 2020; Patel et al., 2021; Li et al., 2022). EDED is distinct from prior ensemble docking approaches, mainly in clustering and selection of receptor models. Global root mean square deviation (RMSD) is convenient to cluster similar structures, but the highly dynamic extracellular and intracellular loops (ECLs and ICLs) of GPCRs can significantly compromise the otherwise good similarity between the 7TM structures. Thus, clustering based on global RMSD can generate many models that, while representative of global changes, are irrelevant to the intricate differences within the local binding pocket of the GPCR. This additional overhead ultimately lowers both the efficiency and accuracy of ensemble docking when using the global RMSD approach for clustering. EDED avoids this issue by focusing on both local similarity and essential dynamics of the binding pocket. Although computational power is more accessible than ever, streamlined workflows which expend computational resources only on worthwhile calculations are always desirable. Herein, we applied EDED to PAC1R with as few as four receptor models, whose results show a reduced false negative rate and a good correlation between the small molecule efficacy and the predicted score. Our results provide the evidence for initial success to develop small-molecule antagonists for PAC1R and pave the way for future structure-based GPCR drug discovery.

## Results and Discussion

### Inactive Conformations of PAC1null and Key Interactions With Small Molecules

Towards discovery of novel PAC1R antagonists, the inactive state conformational ensemble of PAC1R was estimated using an all-atom MD simulation of a ligand-bound PAC1R model from homology modeling (Figure 1). Our reference ligand is an analog of known PAC1 antagonists (Beebe et al., 2008) that were discovered previously using structure activity relationships. We created the antagonist-bound model by docking the reference compound into the PAC1R homology model. This complex model was simulated in the POPC membrane for 500 ns, and for the entire length of the simulation the ligand remained bound in roughly the starting conformation (Supplementary Figure S2). Other features like the closed ECD and straight transmembrane helix six (TM6), as well as short separation between TM3, TM5 and TM6, are consistent with a deactivated structure of a class B GPCR (Wu et al., 2020).

**FIGURE 2:**
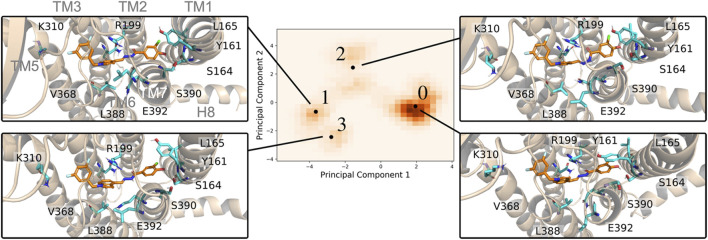
Four representative PAC1R conformations using in EDED reveal important changes in the binding pocket. The protein is shown in cartoon and the reference ligand is shown as sticks. The histogram of all trajectory frames projected onto the first two principal components of residues within the ligand-binding pocket of PAC1R. Black dots labelled with numbers from 0 to 3 are the representative structures (S0, S1, S2, and S3) determined by the *K*-means clustering algorithm.

Despite the overall appearance of an inactivated receptor, there were critical changes within the orthosteric pocket during the MD simulations. Using EDED, four members of the inactive conformational ensemble (states S0, S1, S2, and S3 ordered by observed population) were extracted and reveal distinct conformations of the 7TM helices and different side chain orientations within the binding pocket (Figure 2). For one, bending of TM1 was observed to follow S0 < S2 < S3 < S1, where the most populated state (S0) was the most straightened helix. This correlated with local changes to residues Y161, L165, and S164 on TM1, and most significantly the stiffened TM1 in the S0 state enabled both π-stacking (with Y161) and hydrogen bonding (with S164) interactions. On the other hand, displacement of TM7 in the S1 state relative to S0 caused replacement of the hydrogen bond with S164 in the S0 state with a new hydrogen bond with S390. The interactions between the indole on the ligand and V368, L388, and E392 were modulated between the different receptor states with generally tighter interactions in the S1 and S3 states, in comparison with the S0 and S2 states. In addition, changes in TM5 affected the ability of K310 to form the stable interactions with the electron rich substituents on the ligand in states S0 and S3 which were diminished in the S1 and S2 states. Ultimately, this analysis reveals how EDED is able capture the subtle changes in pocket structure that are highly relevant for accurate modeling of ligand-receptor interactions when performing SBDD.

### Comparison of Docking to a Single Receptor Model and to the Conformational Representatives

**FIGURE 3:**
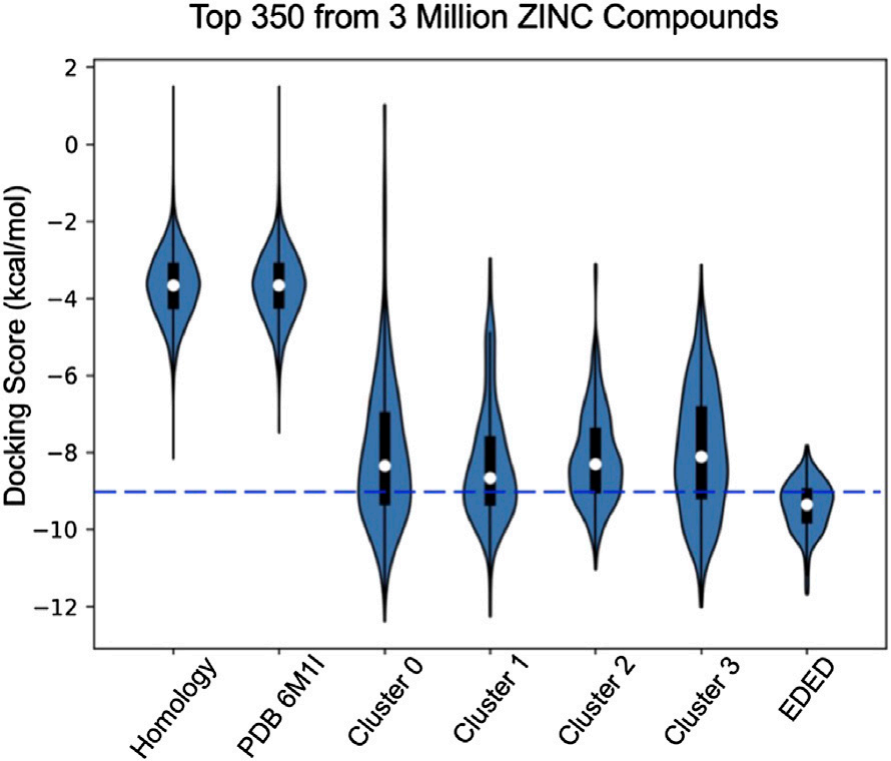
Violin plots of the docking score distribution of the top 350 compounds to different receptor models. The dash line shows the –9.0 kcal/mol cutoff used to prioritize compounds for synthesis.

Compared with docking to the ligand-free homology model and ligand-free cryo-EM structure, EDED significantly improved the identification of candidate compounds (Figure 3). The average binding score of the top 350 (approximately 2.5%) of compounds docked to the ligand-free homology model improved from −5.9 to −9.4 kcal/mol when docked against the ensemble. Likewise, it improved from an average of −5.8 to −9.4 kcal/mol when compared to the PACAP-bound model (PDBID: 6M1I). This gives an average 3.6 kcal/mol improvement in average docking score of the top selected compounds. Additionally, EDED identified six compounds predicted to bind to PAC1R with comparable binding score (−11.2 kcal/mol) as our reference ligand**.**

To gain physical insight into the improvement of the docking scores, the binding pose(s) of the top compounds from both methodologies were examined. We have previously reported the key role of R199 in PACAP-induced activation of PAC1R (Liang et al., 2020). This is further corroborated by strong cation-pi interactions with the residue in our models. Interaction with R199 across all the ensemble conformations became a critical determining factor for which top ensemble docking compounds should be prioritized for synthesis and/or computational optimization. Examining the compounds which have ensemble docking scores close to or better than our reference ligand, this interaction is present for all six top scoring ligands in *at least* one of the docking poses. This is in contrast with the homology model and the PACAP-bound model where only relatively few of the top compounds from this methodology were able to engage in this key interaction. Also, of note are induced fit effects where the MD simulation of our reference ligand in the pocket may affect the binding pocket through subtle shifts in the backbone and the rotation of side chains. In the rigid receptor docking to the homology model, the 7TM helical bundle is closer together, defining a more compact orthosteric pocket. Thus, it is only accessible for small ligands to bind deep into the pocket below R199. In contrast, the conformations in the ensemble docking are more open, better allowing ligands to access the pocket. This can be seen by where most ligands found their best pose. Although both datasets were docked against a grid centered on R199, the ensemble docking results have the majority of top ligands below the residue, low in the pocket. When docked against the homology model, the top ligands are higher in the pocket at lowest in line with R199.

The new ligands examined within the orthosteric pocket showcased the ability of ensemble docking to provide integral confirmations omitted by static modelling, with the ensemble approach providing key ligand poses corresponding to interactions with new side chains revealed in the ensemble. Aside from R199, several key contacts were discovered from study of the top ligands bound to each receptor in the ensemble (Supplementary Figure S3). These contacts expand the understanding of the orthosteric pocket dynamics and can be exploited in small molecule rational design. In comparison with consistent interactions to the ligand-binding pocket of the homology model, these results suggests that EDED may reveal new crucial ligand-receptor interactions even from a rigid template.

A thermodynamically driven approach to scoring the binding poses of a given compound to multiple receptor structures was used to assess the binding affinity of the docked ligands. This approach quantitatively captures various physical phenomena that are often considered when computing overall docking scores: 1) the relative likelihood of the receptor obtaining the different conformations are explicitly included, and 2) the binding of the ligand to the receptor changes the energies of the complex differentially in the distinct conformations. Importantly, this model properly handles confounding cases that other approaches, such as a simple direct averaging of different docking scores, would not describe well. For instance, for any given ligand, a protein is hypothetically able to adopt an unlikely conformation ( $\Delta G{\_}_{\mathrm{conf}1,i}\gg 0$ , i.e., much higher relative free energy than the structure with lowest relative free energy) where the binding of the ligand to the protein could be quite favorable ( $-\Delta G_{bind_{i}}$ approximately equal to 10 kT). Simply including this state in an average of docking scores would treat it as equivalently important as conformations that are far more relevant to the signaling states of the protein. Our approach avoids such errors, by including the energetics of the receptor confirmations, assuring that the overall energy of these rare states is indeed still relatively high and do not contribute significantly to the final score in Eq. 1. In sum, our docking score considers the difference in overall energies of the bound receptor conformations and is appropriate for comparison with a physical experiment that is unlikely to be able to distinguish between different bound conformations (Eq. 1).

$$e^{\Delta G/kT}=e^{\left(G_{bound}-G_{unbound}\right)/kT}=\frac{P_{unbound}}{P_{bound}}=\frac{\sum_{i=1}^{n}P_{cluster\;i}}{\sum_{i=1}^{n}P_{complex\;i}}$$ (1)

Where $G_{bound}$ and $G_{unbound}$ are the energies associated with the ligand being bound or unbound to any receptor conformation, respectively, $P_{bound}$ and $P_{unbound}$ are the total probabilities of the ligand being bound or unbound to any receptor conformation in the ensemble, respectively, $P_{cluster\;i}$ is the probability of a specific receptor conformation (calculated from the MD, see SI for more information), and $P_{\mathrm{complex}\;i}$ is the probability of the ligand being bound to that specific ensemble conformation. We note that our model is still more appropriate than equal weighting for cases where one does not trust the relative energies of the different conformations obtained directly from the MD simulations. In such cases setting the $\Delta G{\_}_{\mathrm{conf}1,i}$ to 0 for each conformation (i.e., each conformation is equally likely) reduces Eqs 1–2.

$$\Delta {\text{G}}_{bind,equal\;weighting}=\ln\left(\frac{n}{\sum_{i=1}^{n}e^{-\Delta G_{bind_{i/kT}}}}\right)kT$$ (2)

Clearly, Eq. 2 is not a simple weighted average of the different binding scores, however to our knowledge this analysis is lacking in the literature.

### Evaluation of EDED Predictions

**FIGURE 4:**
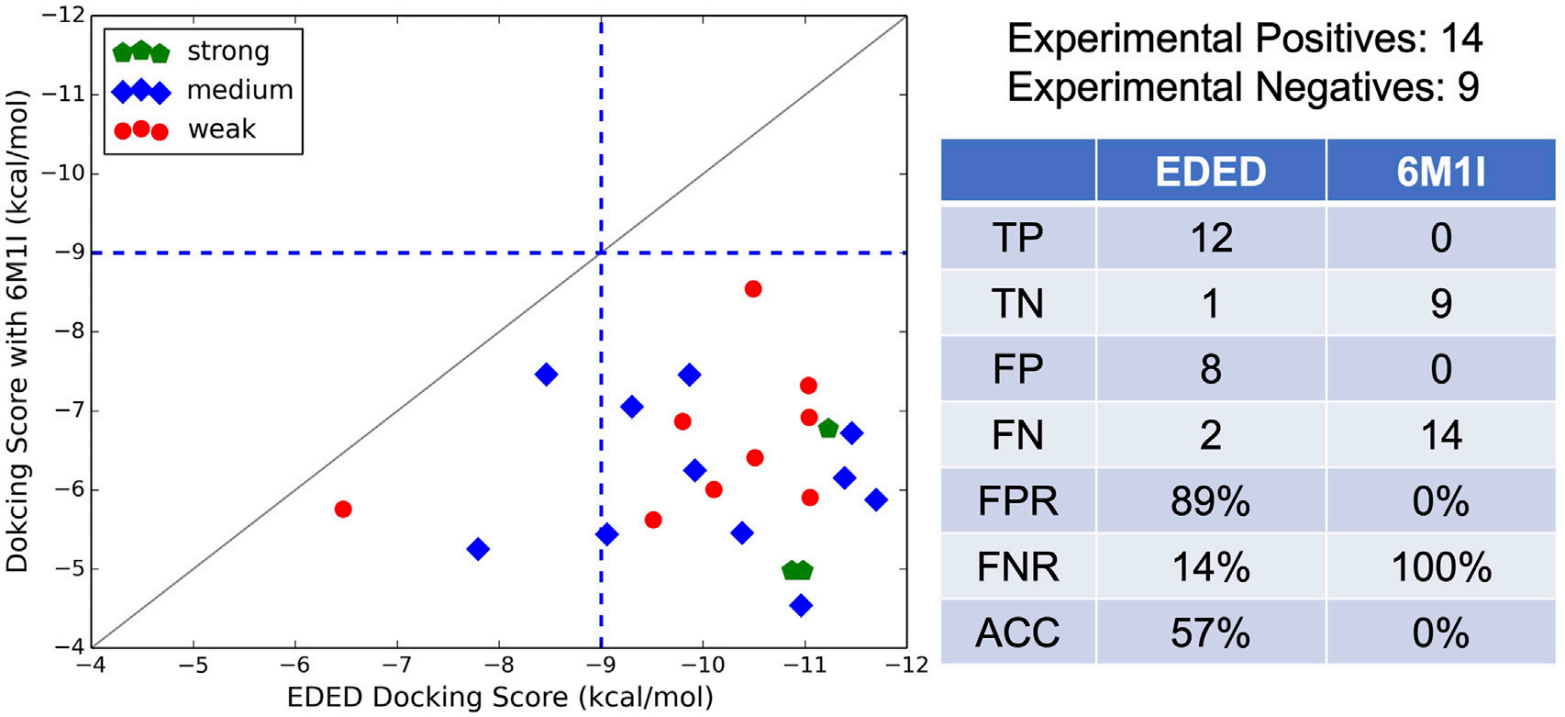
Ensemble weighted glide scores ( Δ G_bind_) of 23 experimentally tested compounds. Compounds with strong, modest, and poor ERK inhibitive activity are depicted in green, blue, and red, respectively. Corresponding colored lines represent the average ensemble weighted glide score for that category. A cutoff of –9 kcal/mol was applied for predicted antagonists to be compared to their experimental results showing either strong or medium inhibition (active) or weak inhibition (inactive).

Additional to testing EDED with compounds from ZINC, we also tested 23 small-molecule compounds which were classified as strong, moderate, and weak antagonists in PAC1R activity assays (unpublished data from Prof. Victor May). The design of these small molecules was based on previously published work outlining the structure-activity relationship between small molecules and the PAC1 receptor (Beebe et al., 2008). Ligand-based virtual screening was then performed and yielded the 23 compounds which were experimentally tested. Docking each analog against all four conformations in the ensemble and scoring them as previous described (Eq. 1) shows modest correlation to experimental results (Figure 4). The strong experimental antagonist had the highest predicted binding affinities with an average −10.4 kcal/mol, while the moderate and weak antagonists both had worse predicted binding affinities −9.8 kcal/mol and −8.5 kcal/mol, respectively.

It is worth noting that our EDED method is best used to identify potential antagonists from a collection of compounds, but the dockings scores (like Glide SP, XP, and our EDED score) to estimate binding energies should be interpreted with caution (Elokely and Doerksen, 2013; Pantsar and Poso, 2018; Pinzi and Rastelli, 2019). While we successfully reduced the false negative rate (FNR) with EDED, there is still a high false positive rate (FPR). A delicate balance between ensemble size and the FPR has previously been reported, inspiring us to select a relatively small ensemble for analysis (Mohammadi et al., 2022). Our FPR is comparable to prior studies employing both ensemble and static methods for virtual screening (Ferreira et al., 2010; Deng et al., 2015; Hou et al., 2018). Additionally, the experimental assays provided here are a measure of antagonistic ability, and not binding affinity. As quantitative binding assays remain to be performed, it is possible some of the false positives (compounds with poor experimental results but high ensemble docking scores) bind tightly but are not effective antagonists, i.e., they do not stabilize the inactive conformations or prevent cognate ligand binding in other ways. With the extended view provided by EDED, we envision that the chance of obtaining a false negative prediction is likely reduced in our model when compared with static Glide docking. This added width within the sampled energy landscape (from the new side chain confirmations) allows our EDED method to achieve more accurate sampling of potential ligand-receptor interactions, thus increasing the chances of finding a hit compound otherwise overlooked in the static model. Overall, EDED displayed an accuracy of 57% in predicted binding affinity when compared to our experimental results, an increase when compared with Glide’s empirical scoring function (Adeshina et al., 2020). Combined with the overall low variance in EDED docking scores for the top 350 compounds analyzed (Figure 3), we believe our methodology represents a robust route for the recognition of small molecules with high receptor affinity.

## Conclusion

In conclusion, we have developed and implemented EDED, an ensemble docking inspired methodology for SBDD. By focusing on the essential dynamics of the ligand binding pocket, our method is distinct from many prior studies that built receptor clusters solely based on the root mean square deviation (RMSD) of the entire protein backbone (Kufareva and Abagyan, 2012). Further, the use of clustering within this reduced dimensionality conformational space directly considers the local structural similarity of the ligand-binding pocket. We demonstrate that EDED captures the critical changes in the 3D structure of the binding pocket that are known to correlate strongly with binding affinity of ligands. Our approach is partially based on the assumption that differences in the binding pocket itself (as opposed to the protein as a whole) predominately give rise to the different binding poses and energies that are the goal of any ensemble docking workflow. Using the EDED derived representative structures, we screened a large dataset of compounds and successfully identified novel small molecule antagonists of the PAC1 receptor. However, EDED is not specific to a single GPCR and will likely accelerate the design of small molecule drugs that target other GPCRs with currently unknown conformational states.

## Methods and Models

### Receptor Model Preparation in EDED

One key idea of EDED is to obtain chemically relevant receptor models for docking. Instead of using the agonist-bound PAC1R structure, we generated a homology model of inactive PAC1R (with the canonical variant sequence, Uniprot ID: P41586) with a template of the glucagon receptor (PDBID: 4L6R, ∼40% similarity) (Boehr et al., 2009). This PAC1R model incorporated the inactive features of class B GPCRs such as a continuous helix along TM6 and a closed ECD. A small-molecule PAC1R antagonist, our reference ligand, was placed in the orthosteric pocket via molecular docking (Glide, Schrödinger Inc.). The complex model was later simulated to sample the inactive conformational ensemble.

### Receptor Model Sampling in EDED

To sample inactive conformations for docking, the ligand-bound PAC1R model was simulated with the OPLS3 (Harder et al., 2016) force field in explicit SPC solvent in the NPT ensemble (300K, 1 atm, Martyna-Tuckerman Klein coupling scheme) using classical MD simulations. A POPC membrane was place around the 7TM using the Orientations of Proteins in Membrane (OPM) database (Lomize et al., 2012). The simulation was performed in the Maestro-Desmond program (Bowers et al., 2006) (GPU version 5.4) with a timestep of 2 fs, recording interval of 4.8 ps, and a total simulation time of 500 ns The Ewald technique was used for the electrostatic calculations. The van der Waals and short-range electrostatic interactions were cut off at 9 Å. Hydrogen atoms were constrained using the SHAKE algorithm. Two extended simulations were also examined to confirm the ligand poses and receptor confirmations. Once again, a POPC membrane was placed around the 7TM bundle using OPM. NAMD 2.11 was used as the simulation package for these replicates (Phillips et al., 2020). The CHARMM36 forcefield (Lee et al., 2016) was used with a TIP3 solvent model in a NPT ensemble (310 K, 1 atm) Force switching was utilized at the range of 10–12 Å to approximate the LJ interactions. Langevin piston/Nose-Hoover (Martyna et al., 1994; Feller et al., 1995) methods were utilized for the pressure control with a piston period of 50 fs and a decay time of 25 fs Langevin coupling of these simulations with a dampening coefficient of 1 ps^−1^ was also utilized. Long range electrostatic interactions were modeled with the particle mesh Ewald method (Essmann et al., 1995). These simulations were run with a 2 fs timestep and combined for 350 ns of data. MD trajectories were analyzed using in-house Python and TCL scripts as well as Visual Molecular Dynamics (VMD). (Humphrey et al., 1996).

### Receptor Ensemble Selection in EDED

**FIGURE 5:**
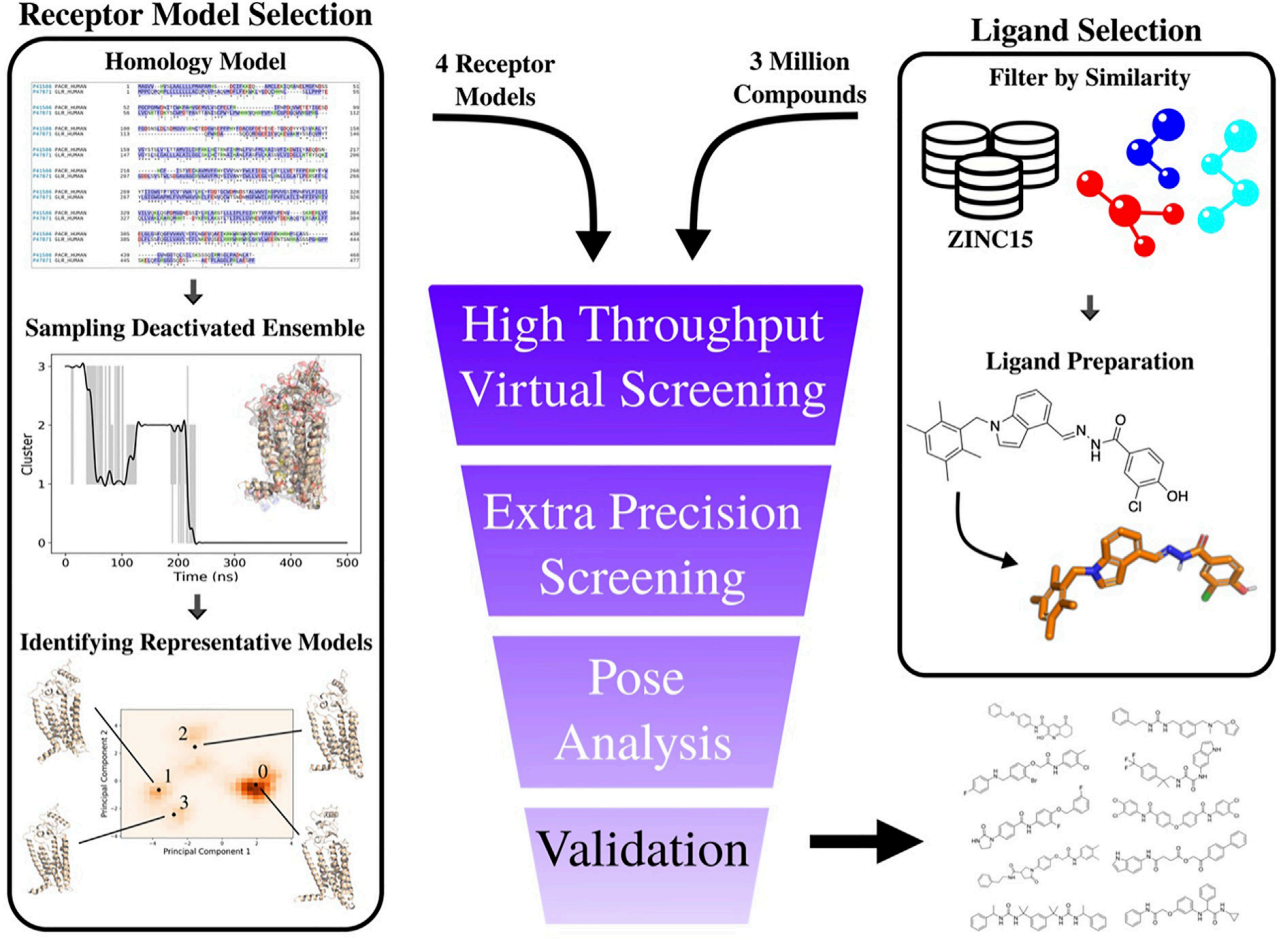
Overview of computational workflow for development of PAC1R antagonists. Right Column: selection of input ligands from a structure database (in this example the ZINC15 (Sterling and Irwin, 2015) database). Custom filters were used to select raw structures with desirable properties (molecular weight, log*P*, etc.). These structures are then prepared using Schrödinger’s *ligprep* software program. Left column): the PAC1null homology model is constructed from the protein’s sequence, simulated for 500 ns, and the raw coordinates are analyzed. The representative structures are used in ensemble docking. Hit compounds are selected based on visual inspection of the results.

We first aligned the 7TM of PAC1R (residues 156–405) to the homology model to reduce noise due to translational movement. Next, the coordinates of the centers of mass for any residue whose side chain was within 3 Å of any ligand atom in the static model were collected and parsed using in-house designed TCL and python scripts. A dimension reduction based on principal component analysis (PCA) was used to determine which collective motions (termed principal components, PCs) contributed most to variations in the overall conformations of the binding pocket. The first fifteen PCs (accounting for 90% of the cumulative variance) were clustered using a *K*-means clustering algorithm implemented by PyEmma (Liao et al., 2021). Based on inspection of the first two PCs (Figure 5), four cluster centers were identified. As these cluster centers are not precise frames within the trajectory but are instead points in the PC space, the cluster centers’ PC coordinates were approximately projected back to the original Cartesian coordinates. Frames from the trajectory which had PC values closest to the centers based on a RMSD measurement, were then selected as the ensemble docking receptor structures. This approach allowed a minimum of representative frames to capture the most variance of the binding pocket as opposed to other methodologies which often have many structures. Also, our physics-based approach is transferrable to other GPCRs and expanded clustering. In fact, our focus on the relevant receptor models likely requires less sampling in MD simulations and fewer clusters for subsequent docking, a practical advantage for large-scale screening.

### Docking and Scoring of Potential PAC1R Antagonists

Receptor grid models were generated using the three-dimensional structures selected as detailed above with R199 selected as the center of the docking box with an 18-Å cutoff. Docking was carried out using Schrödinger Virtual Screening Workflow (Friesner et al., 2006) (VSW) at three consecutive levels of precision, both for small molecules docked to the static homology model and to the conformation ensemble. Small molecules docked to our PAC1null ensembled were given an overall score, Ensemble Δ G_bind_, based on Eq. 3.

$$\text{Ensemble }\Delta {\text{G}}_{bind}=\ln\left(\frac{1+\sum_{i=2}^{n}e^{-\Delta G_{conf_{1,i}}}}{\sum_{i=1}^{n}e^{-\Delta G_{conf_{\left(1,i\right)}}-\Delta G_{bind_{(1,i)/kT}}}}\right)kT$$ (3)

In Eq. 3, $\Delta G_{\mathrm{conf}1,i}$ is the difference in energy (in units of kT) between the lowest energy receptor conformation and each subsequent conformation calculated using the clustered trajectory, and $-\Delta G_{bind_{i}}$ is the corresponding Glide XP docking score to that same conformation. While $\Delta G_{\mathrm{conf}1,i}$ is representative of the apo receptor free energy, it is worth noting that simulation data used to generate these confirmations included the ligand bound within the pocket.

Docking was carried out against compounds 1) pseudo-randomly selected from the ZINC15 (Sterling and Irwin, 2015) database, 2) as analogs of known antagonists to the static ligand-free homology model, the cryo-EM structure, and the conformational ensemble. In total, a small test set of 10,000 drug-like compounds were selected and download from the ZINC database and docked using Schrödinger’s VSW as described previously.

## Data Availability

The raw data supporting the conclusion of this article will be made available by the authors, without undue reservation.
