# Supplementary material for: Essential Dynamics Ensemble Docking for Structure-Based GPCR Drug Discovery
Source: Front Mol Biosci. 2022 Jun 29;9:879212. doi: 10.3389/fmolb.2022.879212 (PMC9277106; doi:10.3389/fmolb.2022.879212)
Supplement: Supplementary file 1 [file DataSheet1.PDF]

Supporting Information:

Essential Dynamics Ensemble Docking for Structure-Based GPCR Drug Discovery

Kyle McKay[#], Nicholas B. Hamilton[#], Jacob M. Remington, Severin T. Schneebeli, Jianing Li*

Department of Chemistry, University of Vermont, Burlington, VT 05405

[#] Equal contributions
*Email: Jianing Li (jianing.li@uvm.edu)

```
P41586     PACR_HUMAN         1  MAGVVHVSLAALLLLPMAPAMHSDCIFKKEQAMCLEKIQRANELMGFNDSSPGCPGMWDN     60
P41586-2   PACR_HUMAN         1  MAGVVHVSLAALLLLPMAPAMHSDCIFKKEQAMCLEKIQRANELMGFNDSSPGCPGMWDN     60
P41586-3   PACR_HUMAN         1  MAGVVHVSLAALLLLPMAPAMHSDCIFKKEQAMCLEKIQRANELMGFNDSSPGCPGMWDN     60
                                 ************************************************************

P41586     PACR_HUMAN        61  ITCWKPAHVGEMVLVSCPELFRIFNPDQVWETETIGESDFGDSNSLDLSDMGVVSRNCTE    120
P41586-2   PACR_HUMAN        61  ITCWKPAHVGEMVLVSCPELFRIFNPDQVWETETIGESDFGDSNSLDLSDMGVVSRNCTE    120
P41586-3   PACR_HUMAN        61  ITCWKPAHVGEMVLVSCPELFRIFNPDQ---------------------DMGVVSRNCTE     99
                                 ****************************                     ***********

P41586     PACR_HUMAN       121  DGWSEPFPHYFDACGFDEYESETGDQDYYYLSVKALYTVGYSTSLVTLTTAMVILCRFRK    180
P41586-2   PACR_HUMAN       121  DGWSEPFPHYFDACGFDEYESETGDQDYYYLSVKALYTVGYSTSLVTLTTAMVILCRFRK    180
P41586-3   PACR_HUMAN       100  DGWSEPFPHYFDACGFDEYESETGDQDYYYLSVKALYTVGYSTSLVTLTTAMVILCRFRK    159
                                 ************************************************************

P41586     PACR_HUMAN       181  LHCTRNFIHMNLFVSFMLRAISVFIKDWILYAEQDSNHCFISTVECKAVMVFFHYCVVSN    240
P41586-2   PACR_HUMAN       181  LHCTRNFIHMNLFVSFMLRAISVFIKDWILYAEQDSNHCFISTVECKAVMVFFHYCVVSN    240
P41586-3   PACR_HUMAN       160  LHCTRNFIHMNLFVSFMLRAISVFIKDWILYAEQDSNHCFISTVECKAVMVFFHYCVVSN    219
                                 ************************************************************

P41586     PACR_HUMAN       241  YFWLFIEGLYLFTLLVETFFPERRYFYWYTIIGWGTPTVCVTVWATLRLYFDDTGCWDMN    300
P41586-2   PACR_HUMAN       241  YFWLFIEGLYLFTLLVETFFPERRYFYWYTIIGWGTPTVCVTVWATLRLYFDDTGCWDMN    300
P41586-3   PACR_HUMAN       220  YFWLFIEGLYLFTLLVETFFPERRYFYWYTIIGWGTPTVCVTVWATLRLYFDDTGCWDMN    279
                                 ************************************************************

P41586     PACR_HUMAN       301  DSTALWWVIKGPVVGSIMVNFVLFIGIIVILVQKLQSPDMGGNESSIY------------    348
P41586-2   PACR_HUMAN       301  DSTALWWVIKGPVVGSIMVNFVLFIGIIVILVQKLQSPDMGGNESSIYFSCVQKCYCKPQ    360
P41586-3   PACR_HUMAN       280  DSTALWWVIKGPVVGSIMVNFVLFIGIIVILVQKLQSPDMGGNESSIY------------    327
                                 ************************************************

P41586     PACR_HUMAN       349  ----------------LRLARSTLLLIPLFGIHYTVFAFSPENVSKRERLVFELGLGSFQ    392
P41586-2   PACR_HUMAN       361  RAQQHSCKMSELSTITLRLARSTLLLIPLFGIHYTVFAFSPENVSKRERLVFELGLGSFQ    420
P41586-3   PACR_HUMAN       328  ----------------LRLARSTLLLIPLFGIHYTVFAFSPENVSKRERLVFELGLGSFQ    371
                                                 ********************************************

P41586     PACR_HUMAN       393  GFVVAVLYCFLNGEVQAEIKRKWRSWKVNRYFAVDFKHRHPSLASSGVNGGTQLSILSKS    452
P41586-2   PACR_HUMAN       421  GFVVAVLYCFLNGEVQAEIKRKWRSWKVNRYFAVDFKHRHPSLASSGVNGGTQLSILSKS    480
P41586-3   PACR_HUMAN       372  GFVVAVLYCFLNGEVQAEIKRKWRSWKVNRYFAVDFKHRHPSLASSGVNGGTQLSILSKS    431
                                 ************************************************************

P41586     PACR_HUMAN       453  SSQIRMSGLPADNLAT                                                468
P41586-2   PACR_HUMAN       481  SSQIRMSGLPADNLAT                                                496
P41586-3   PACR_HUMAN       432  SSQIRMSGLPADNLAT                                                447
                                 ****************
```

Figure S1. Protein Sequence of PAC1null (Uniprot ID: P41586 or P41586-1) and PAC1 very short (Uniprot ID: P41586-3). ECD: residues 21-155 and 7TM: residues 156-468. The 21-amino acid ECD insert is highlighted with the yellow background.

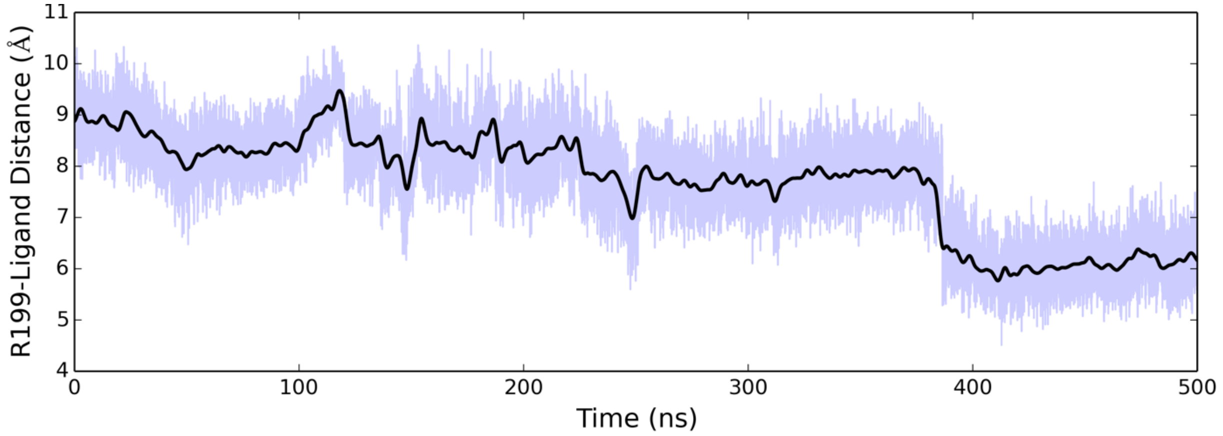

Figure S2. Center-of-Mass (COM) Distance between our reference ligand and PAC1null R199 over the course of 500 ns of simulation data. Four microstates are available with the timeframe, with the ligand remaining bound in the orthosteric pocket for the duration of simulation. An average distance of 7.7 Å was found between the ligand and R199.

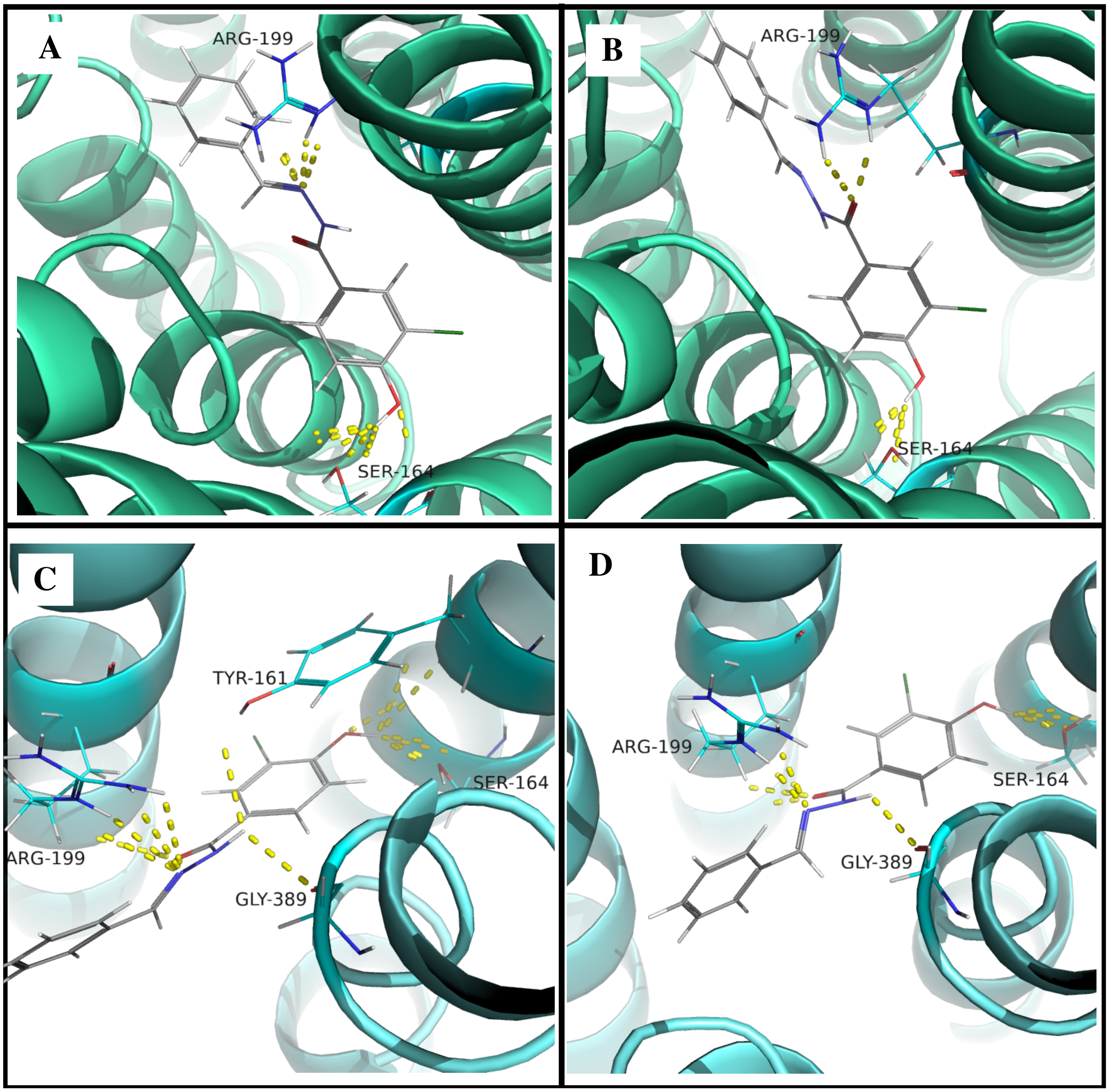

Figure S3. Comparison of ensemble receptor model binding pockets with ligand confirmation. One ligand is shown in grey with polar contacts showcased with R199, S164, Y161, and G389. G389 represents a new, ligand-receptor contact within the orthosteric pocket, outside of those found for the reference ligand. Other top scoring ligands also revealed new unique side chain reactions deeper within the pocket.
